# Serum Procalcitonin and Presepsin Levels in Patients with Generalized Pustular Psoriasis

**DOI:** 10.1155/2018/9758473

**Published:** 2018-12-16

**Authors:** Makoto Nagai, Yasutomo Imai, Yoshihiro Wada, Minori Kusakabe, Kiyofumi Yamanishi

**Affiliations:** Department of Dermatology, Hyogo College of Medicine, 1-1, Mukogawa-cho, Nishinomiya, Hyogo 663-8501, Japan

## Abstract

Patients with generalized pustular psoriasis (GPP) often present with symptoms that must be differentiated from sepsis. Procalcitonin (PCT) and presepsin (P-SEP) are widely used as biomarkers for sepsis; therefore, we examined the serum PCT and P-SEP levels in patients with psoriatic diseases. The enrolled patients included 27 with psoriasis vulgaris (PV) (22 males, 5 females; mean age 47.7 years), 12 with psoriatic arthritis (PsA) (8 males, 4 females; mean age 51.3 years), and 15 with GPP (10 males, 5 females; mean age 63.7 years). The mean serum PCT levels in patients with PV, PsA, and GPP were 0.01 ng/mL (25th–75th percentile; 0.00–0.03), 0.013 ng/mL (0.00–0.03), and 0.12 ng/mL (0.05–0.18), respectively; the levels of PCT were higher for patients with GPP than with PV or PsA but were lower than the PCT cutoff value (0.5 ng/mL) for the diagnosis of infection. The mean serum P-SEP levels in patients with PV, PsA, and GPP were 144.9 pg/mL (25th–75th percentile; 78–181), 168.1 pg/mL (124–203), and 479.9 pg/mL (216–581), respectively. Unexpectedly, the levels of P-SEP in the patients with GPP were as high as the P-SEP cutoff value (317 to 647 pg/mL) used for the diagnosis of infection. We also found that neutrophils produced P-SEP, suggesting that the high serum P-SEP levels in patients with GPP might arise at least in part due to the P-SEP derived from neutrophils activated in GPP. Both serum PCT and P-SEP might therefore be useful as novel serum biomarkers for GPP because their levels were decreased by GPP treatments. However, the measurement of PCT might be more useful than the measurement of P-SEP for discriminating between GPP and sepsis.

## 1. Introduction

Procalcitonin (PCT) is a 116-amino-acid precursor peptide of the hormone calcitonin (CT) [1]. In noninfected conditions, PCT is mainly produced as a precursor of CT by the C cells of the thyroid gland. However, PCT can also be produced from nonneuroendocrine tissues due to infections, including sepsis, which contribute to increase in blood PCT levels [2]. Numerous studies on PCT have been published since the ‘90s, and PCT is now widely used as a biomarker for bacterial infection or sepsis [3].

Presepsin (P-SEP), a soluble CD14 subtype, is also now recognized as a new sepsis biomarker. P-SEP is a small 13 kDa protein that arises due to cleavage of the N-terminal fragment of CD14 by elastase [4]. Membrane-bound CD14 has a high affinity for bacterial lipopolysaccharide and is expressed on the cell surface of monocytes, macrophages, and neutrophils. Arai et al. reported that monocytes were the main source of P-SEP in humans and that P-SEP secretion by monocytes is triggered by phagocytosis rather than by soluble inflammatory stimuli [5]. Compared with other sepsis markers, including C-reactive protein (CRP) and PCT, P-SEP appears to have a better specificity and sensitivity for the diagnosis of sepsis [4], as blood levels of P-SEP in patients with injuries or burns are almost normal [6].

Psoriasis is a chronic inflammatory skin disease that has several forms [7]. The major form of this disease is psoriasis vulgaris (PV), which is characterized by erythematous plaques with thick scales. Another commonly encountered form is psoriatic arthritis (PsA), a seronegative arthropathy that is associated with psoriatic skin lesions and characterized by chronic arthritis, dactylitis, and enthesitis. By contrast, GPP is a rarer but more severe subtype of psoriasis and is characterized by multiple sterile pustules that develop all over the body surface [7–9]. Unlike PV or PsA, GPP is associated with a generalized erythematous flush and sepsis-like systemic symptoms, including high fever, leukocytosis, and elevated CRP levels. The severe and intractable inflammatory conditions associated with GPP, which include leukocyte activation, mean that therapy for GPP is typically leukocytapheresis or granulocyte and monocyte apheresis, in addition to administration of methotrexate, cyclosporine, prednisolone, or biologics such as the anti-TNF-alpha antibody infliximab or the anti-IL-17 antibody secukinumab [7, 9].

Psoriasis is possibly triggered by activation of cytokines, such as TNF-alpha, IL-17, IL-12, and IL-23; therefore, these cytokines are now therapeutic targets for the disease [8]. However, considering the sepsis-like manifestations in GPP, we speculated that clinical tests for PCT and P-SEP, the markers of sepsis, might also be candidate biomarkers for GPP. In this study, we examined the serum levels of PCT and P-SEP in psoriatic diseases, and we propose that PCT, but possibly not P-SEP, may be a useful biomarker for GPP.

## 2. Patients and Methods

### 2.1. Study Subjects

The enrolled patients included 27 with PV, 12 with PsA, and 15 with GPP (Table 1) treated at the Hyogo College of Medicine Hospital, Hyogo, Japan, from January 2006 to December 2017. This study was approved by the Ethics Review Board of Hyogo College of Medicine (no. 212), and informed consent was obtained from all patients. Blood was collected at the baseline or at the first visit (before therapy) and again at follow-ups after treatment, but blood sampling was not necessarily scheduled because additional samplings of human materials, except for clinical need, were not permitted by the Ethics Review Board.

### 2.2. Measurement of PCT and P-SEP

PCT concentrations were measured using a Brahms procalcitonin (PCT) kit (Wako, Osaka, Japan), and P-SEP concentrations were measured with a chemiluminescence enzyme immunoassay (CLEIA) presepsin kit (LSI Medience, Tokyo, Japan) [10]; all procedures were conducted according to the manufacturers' instructions. The blood samples were analyzed by SRL Kobe Laboratory (SRL Inc., Japan).

### 2.3. Isolation and Stimulation of Monocytes and Neutrophils

Monocytes were isolated from human whole blood from healthy volunteers using StraightFrom™ Whole Blood CD14 MicroBeads and a Monocyte Isolation Whole Blood Column Kit (Miltenyi Biotec, Bergisch Gladbach, Germany), while neutrophils were isolated using a MACSxpress Neutrophil Isolation Kit (Miltenyi Biotec); all procedures were conducted according to the manufacturers' instructions. Residual erythrocytes were lysed using ACK Lysing Buffer (Thermo Fisher Scientific, Waltham, MA). The purity of the enriched CD14(+) monocytes (>90%) was evaluated using an anti-CD14 antibody (BD Biosciences, San Jose, CA), and the purity of the CD16(+) neutrophils (>97%) was analyzed using an anti-CD16 antibody (BD Biosciences); all cells were characterized by flow cytometry using an LSR-II instrument (BD Biosciences). Monocytes (2 × 10^6^ cells per mL) or neutrophils (5 × 10^6^ cells per mL) were cultured for 5 hours in RPMI 1640 medium containing 10% fetal bovine serum in a 48-well plate (IWAKI, Tokyo, Japan), with or without addition of 0.5 mg/mL of pHrodo Green *E. coli* BioParticles (Thermo Fisher Scientific). The concentration of P-SEP in the culture medium was then measured.

### 2.4. Statistics

Data were analyzed using GraphPad Prism version 6 (GraphPad Software Inc., San Diego, CA). Tukey's multiple comparison test was used for multiple comparisons. A two-tailed unpaired *t*-test was used for single comparisons. The Wilcoxon matched-pairs signed-rank test was used for intragroup comparison. Correlations were calculated using Spearman's test. A *p* value less than 0.05 was considered statistically significant.

## 3. Results

The enrolled patients included 27 with PV (22 males, 5 females; mean age 47.7 years, range 18–85), 12 with PsA (8 males, 4 females; mean age 51.3 years, range 35–74), and 15 with GPP (10 males, 5 females; mean age 63.7 years, range 36–92) (Table 1).

### 3.1. Serum PCT Levels in Patients with GPP

**Figure 1:**
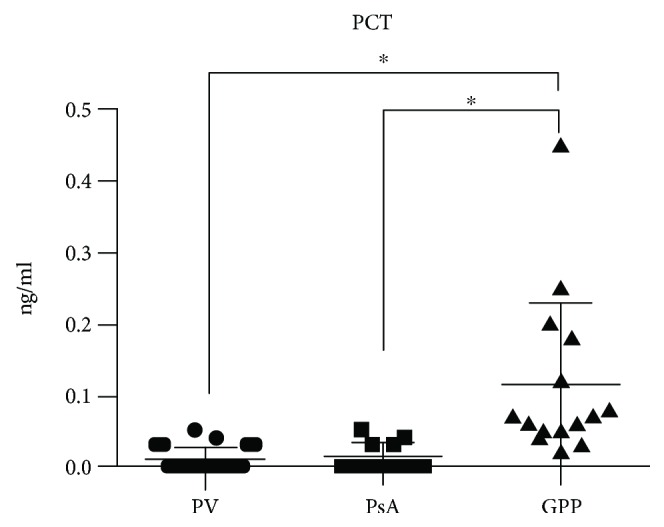
Serum procalcitonin (PCT) levels in the study patients with generalized pustular psoriasis (GPP), showing the serum PCT levels in patients with psoriasis vulgaris (PV), psoriatic arthritis (PsA), and GPP at the first visit. Values are expressed as means (horizontal lines) and standard deviations (top and bottom bars). Note that the PCT concentrations are typically below 0.05 ng/mL in healthy people, and a cutoff value of 0.5 ng/mL is widely used for an appropriate indicator of infection. Tukey's multiple comparison test, ^∗^*p* < 0.001.

Serum PCT levels (at the first visit) were increased in patients with GPP than in patients with PV (Figure 1); the mean serum PCT levels in patients with PV, PsA, and GPP were 0.01 ng/mL (25th–75th percentile; 0.00–0.03), 0.013 ng/mL (0.00–0.03), and 0.12 ng/mL (0.05–0.18), respectively. The serum PCT of healthy individuals is typically below the limit of detection in clinical assays (0.01 ng/mL), so levels less than 0.15 ng/mL make the diagnosis of significant bacterial infection “unlikely” [11], whereas levels greater than the cutoff level 0.5 ng/mL are considered appropriate for the diagnosis of infection [12, 13]. Serum PCT levels in patients with GPP were lower than the cutoff level.

### 3.2. Serum P-SEP Levels in Patient with GPP

**Figure 2:**
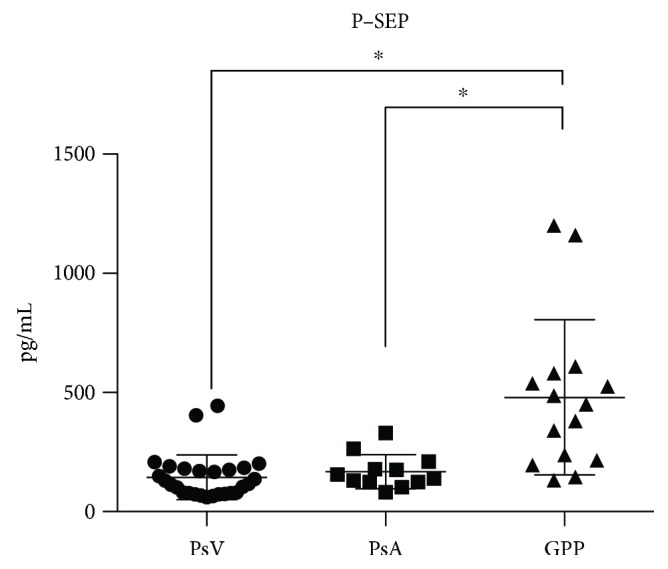
Serum presepsin (P-SEP) levels in patients with psoriasis vulgaris (PV), psoriatic arthritis (PsA), and generalized pustular psoriasis (GPP) at the first visit. Values are expressed as means (horizontal lines) and standard deviations (top and bottom bars). Note that P-SEP concentrations are typically below 314 pg/mL in healthy people, and a cutoff value of 500 pg/mL is widely used for an appropriate indicator of infection. Tukey's multiple comparison test, ^∗^*p* < 0.001.

Serum P-SEP levels (at the first visit) were significantly increased in patients with GPP (Figure 2) when compared to patients with either PV or PsA; the mean serum P-SEP levels in patients with PV, PsA, and GPP were 144.9 pg/mL (25th–75th percentile; 78–181), 168.1 pg/mL (124–203), and 479.9 pg/mL (216–581), respectively. The normal range of P-SEP in healthy adults is reported as 155 pg/mL (25th–75th percentile; 109–231), and the optimal cutoff values for diagnosing sepsis by CLEIA range from 317 to 647 pg/mL [10]. We found that serum P-SEP levels in patients with GPP were as high as those reported in patients with sepsis.

### 3.3. Correlations of Serum CRP, PCT, and P-SEP in Patients with GPP

**Figure 3:**
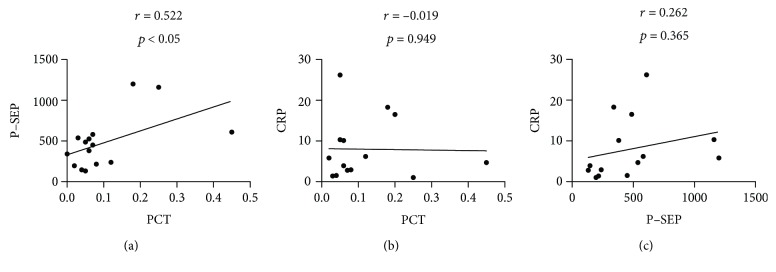
Correlations of serum procalcitonin (PCT), presepsin (P-SEP), and C-reactive protein (CRP). Correlations between serum PCT and P-SEP (a), between PCT and CRP (b), and between PCT and CRP (c).

Because serum CRP levels were elevated in patients with GPP, we measured the serum levels of CRP to determine any possible correlations with serum PCT or P-SEP levels. A significant correlation was found between serum PCT and P-SEP levels (*r* = 0.522, *p* < 0.05) (Figure 3(a)), whereas no significant correlation was noted between serum CRP levels and either serum PCT or P-SEP levels (Figures 3(b) and 3(c)).

### 3.4. Serum PCT and P-SEP Levels after Treatment of GPP

**Figure 4:**
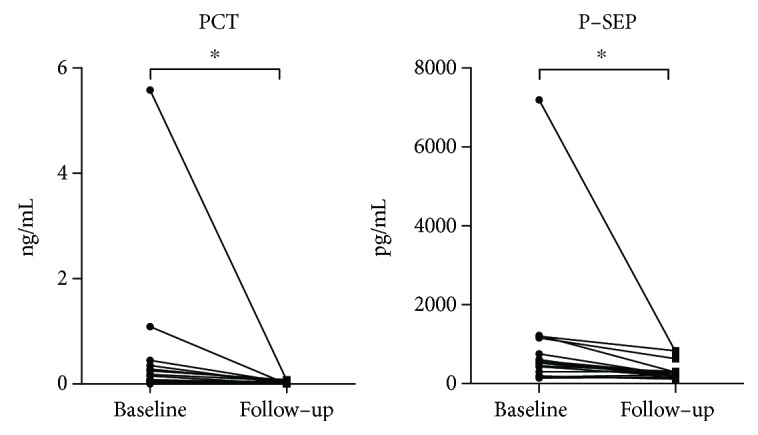
The high serum procalcitonin (PCT) and presepsin (P-SEP) levels in patients with generalized pustular psoriasis (GPP) were significantly decreased after treatment. The serum PCT and P-SEP levels before and after the treatment in 15 patients with GPP are shown. Wilcoxon matched-pairs signed-rank test, ^∗^*p* < 0.001. Characteristics of patients are described in Table 2.

The characteristics of patients with GPP and the details of their treatment are shown in Table 2. The high serum PCT and P-SEP levels in 15 patients with GPP before the treatment (the acute exacerbation period) were significantly decreased after treatment (Figure 4).

### 3.5. Neutrophils Produce P-SEP

**Figure 5:**
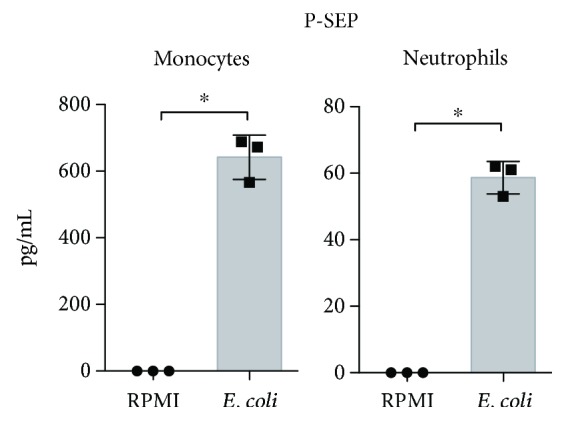
Neutrophils produce presepsin (P-SEP). Monocytes or neutrophils from healthy volunteers were cultured with (*E. coli*) or without (RPMI) pHrodo *E. coli* particles, and the concentrations of P-SEP in the culture media were measured. Values are expressed as means (column graph) and standard deviations (top and bottom bars), *n* = 3. ^∗^*p* < 0.05 (two-tailed unpaired *t*-test). Data are representative of at least two independent experiments.

Monocytes have recently been identified as the main source of P-SEP, and P-SEP secretion is triggered by phagocytosis rather than soluble inflammatory stimuli [5]. However, neutrophils also play a central role in the pathophysiology of GPP [14], so we hypothesized that neutrophils, as well as monocytes, could produce P-SEP. Indeed, P-SEP production occurred in monocytes as well as in neutrophils isolated from human whole blood from healthy volunteers following stimulation with pHrodo *E. coli* particles (Figure 5).

## 4. Discussion

The diagnosis of GPP is sometimes difficult, and infection or sepsis should be considered in the differential diagnosis of patients with GPP and high fever [8, 14].

The PCT algorithm in patients with sepsis either encourages (>0.5 ng/mL) [13] or discourages (<0.25 ng/mL) the initiation of antibiotic therapy by monitoring PCT kinetics [12]. In the present study, the serum PCT levels were slightly increased in the patients with GPP (Figure 1), but the mean serum PCT levels in these patients (0.12 ng/mL) were not particularly high; therefore, microbial infection was considered “unlikely.”

Measurement of serum P-SEP levels is valuable for the very early diagnosis of sepsis arising from bacterial or fungal infections [15] because rises in P-SEP levels occur 12 to 48 hours earlier than rises are observed in the levels of other biomarkers (e.g., PCT and CRP). Surprisingly, the mean serum P-SEP levels were very high (479.9 pg/mL) in patients with GPP (Figure 2), and this high level might be misinterpreted as a “likely” microbial infection, given that the optimal cutoff value for P-SEP in the diagnosis of sepsis is about 500 pg/mL [10]. Therefore, P-SEP might not be of particular value for the differential diagnosis of GPP and sepsis in patients with fever.

Serum PCT levels show a positive correlation with CRP levels in patients with bacterial infection [4, 13]. Conversely, no significant correlations were evident between CRP and PCT or P-SEP levels (Figure 3), which may reflect the fact that GPP is not an infectious disease.

The elevated serum PCT and P-SEP levels in patients with GPP were significantly lowered after GPP treatment (Figure 4). Therefore, PCT and P-SEP could both be useful as novel serum biomarkers for GPP.

PCT has been documented as a marker of bacterial infection. However, PCT also mediates the inflammatory response; for example, high serum PCT levels have been reported in patients with Kawasaki disease [16] or with invasive trauma [4]. By contrast, the values of P-SEP do not show a similar elevation, even in patients with trauma [4]. In this respect, P-SEP, unlike PCT, appears to be highly disease specific. Therefore, the finding of very high serum P-SEP levels in patients with GPP was surprising (Figure 2).

P-SEP is a truncated fragment of the CD14 N-terminal, and both monocytes and neutrophils express CD14 on their membranes [17]. The production of P-SEP by monocytes, but not by neutrophils, has recently been reported in response to *E. coli* particles [5]. The incubation time of the cells with *E. coli* particles in the previous report was 3 hours, but we detected P-SEP production by neutrophils after 5 hours of incubation (Figure 5). Because neutrophils have a known involvement in the disease process of GPP [14], our data suggest that the high serum P-SEP levels observed in patients with GPP might arise at least partly from P-SEP derived from neutrophils.

GPP is an autoinflammatory skin disorder, and infection is only one of the triggering factors for GPP. Even after the triggering factors have diminished, patients with GPP continue to show generalized inflammatory conditions, such as multiple sterile pustules with neutrophil infiltrates in the skin, fever, and increases in CRP [5]. The observed high serum P-SEP levels in GPP might reflect a sterile activation of P-SEP-producing cells, such as monocytes and/or neutrophils. However, further study is required to elucidate mechanisms responsible for the increase in serum P-SEP levels.

This study has some limitations. In particular, GPP is very rare disease, so enrolment of large numbers of patients was difficult.

Taken together, our findings suggest that P-SEP might not be useful for distinguishing between GPP and sepsis, because the P-SEP levels in patients with GPP were as high as the cutoff level for sepsis. In conclusion, we propose that the measurement of PCT levels could be useful for discriminating between GPP and sepsis.

## Figures and Tables

**Table 1:** Characteristics of the study patients.

	PV	PsA	GPP	*p* value		
				Controls vs. PV	Controls vs. GPP	PV vs. GPP
Patients, no.	27	12	15			
Male sex, no. (%)	22 (81.5)	8 (66.6)	10 (66.6)	*p* > 0.05	*p* > 0.05	*p* > 0.05
Age (years)						
Mean	47.7	51.3	63.7	*p* > 0.05	*p* > 0.05	*p* > 0.05
Range	18–85	35–74	36–92			

PV: psoriasis vulgaris; PsA: psoriatic arthritis; GPP: generalized pustular psoriasis.

**Table 2:** Characteristics of the study patients with generalized pustular psoriasis (GPP), showing the serum inflammatory marker levels before and after treatment in 15 patients.

Age (years)	Sex	PCT (ng/mL)		P-SEP (pg/mL)		CRP (mg/dL)		Treatment
		Baseline	Follow-up	Baseline	Follow-up	Baseline	Follow-up	
76	M	0.07	<0.02	581	269	6.2	0.7	Etretinate
73	M	5.58	0.07	7190	807	23.7	4.5	LCAP
55	M	1.09	<0.02	1220	291	7.7	0.1	MTX+CyA
70	M	0.03	<0.02	538	267	4.7	0.1	Etretinate+PSL
62	F	0.45	0.03	609	120	26.2	0.3	MTX
71	M	0.06	0.03	526	209	4.4	1.2	PSL
67	M	0.07	0.04	757	210	14.5	0.2	IFX
54	F	0.35	<0.02	301	125	22.2	0.2	IFX
55	M	0.18	0.06	1200	834	5.8	1.7	MTX+CyA
57	M	<0.02	<0.02	195	116	1	0.1	GMA
62	M	0.25	0.08	1160	631	10.3	0.2	CyA
92	M	0.28	0.04	173	223	0.1	0.2	GMA
78	F	0.07	0.03	450	308	1.5	0.5	GMA
52	F	0.04	0.03	146	162	3.9	0.1	PSL
36	F	0.08	0.03	434	192	8.15	0.15	SEC

PCT: procalcitonin; P-SEP: presepsin; CRP: C-reactive protein; LCAP: leukocytapheresis; MTX: methotrexate; CyA: cyclosporine; PSL: prednisolone; IFX: infliximab; GMA: granulocyte and monocyte apheresis; SEC: secukinumab.

## Data Availability

No data were used to support this study.
